# Functional Validation of *Glutamine* *synthetase* and *Glutamate* *synthase* Genes in Durum Wheat near Isogenic Lines with QTL for High GPC

**DOI:** 10.3390/ijms21239253

**Published:** 2020-12-04

**Authors:** Domenica Nigro, Stefania Fortunato, Stefania Lucia Giove, Elisabetta Mazzucotelli, Agata Gadaleta

**Affiliations:** 1 Department of Soil, Plant and Food Sciences, University of Bari Aldo Moro, 70126 Bari, Italy; 2 Department of Biology, University of Bari Aldo Moro, 70126 Bari, Italy; stefania.fortunato26@gmail.com; 3 Department of Agricultural and Environmental Science, University of Bari Aldo Moro, 70126 Bari, Italy; stefanialucia.giove@uniba.it; 4 CREA—Research Centre for Genomics and Bioinformatics, 29017 Fiorenzuola d’Arda, Italy; elisabetta.mazzucotelli@crea.gov.it

**Keywords:** near isogenic lines, durum wheat, grain protein content, QTL, glutamine synthetase, glutamate synthase

## Abstract

Durum wheat (*Triticum turgidum* L. ssp. *durum*) is a minor crop grown on about 17 million hectares of land worldwide. Several grain characteristics determine semolina’s high end-use quality, such as grain protein content (GPC) which is directly related to the final products’ nutritional and technological values. GPC improvement could be pursued by considering a candidate gene approach. The glutamine synthetase (GS)/glutamate synthase (GOGAT) cycle represents a bottleneck in the first step of nitrogen assimilation. QTL for GPC have been located on all chromosomes, and several major ones have been reported on 2A and 2B chromosomes, where *GS2* and *Fd-GOGAT* genes have been mapped. A useful and efficient method to validate a putative QTL is the constitution of near-isogenic lines (NILs) by using the marker found to be associated to that QTL. Here, we present the development of two distinct sets of heterogeneous inbred family (HIF)- based NILs segregating for *GS2* and *Fd-GOGAT* genes obtained from heterozygous lines at those loci, as well as their genotypic and phenotypic characterizations. The results allow the validation of the previously identified GPC QTL on 2A and 2B chromosomes, along with the role of these key genes in GPC control.

## 1. Introduction

Durum wheat (*Triticum turgidum* var. *durum* Desf.) represents about 5% of the global wheat production and is mainly grown in three principal areas: the Mediterranean basin, the Northern Plains between USA and Canada, and within the desert areas of South West USA and Northern Mexico, with a global production which exceeded 38 million tons in the last cropping seasons. Among the Mediterranean countries, Italy is the major durum wheat producer with an annual average of almost 4.0 MMT (million metric tons) (International Grain Council, https://www.igc.int/en/default.aspx). Despite several local food products are obtained from durum wheat semolina (the coarse, purified durum wheat middlings), such as typical breads, couscous, or bulgur, this cereal crop is mainly used for high-quality pasta production. One of the most important characteristics determining semolina’s high end-use quality is grain protein content (GPC), as directly related to both nutritional and technological values of final food products [1,2]. The development of high GPC new varieties has been a constant priority in breeding programs, although it has been difficult to pursue, as GPC is a quantitative trait, regulated by a complex genetic system and affected by environmental factors, as well as genotype and management practices [3]. Moreover, one relevant aspect to be considered in GPC breeding programs is the well-known strong negative correlation with grain yield, which makes the simultaneous increase of both traits challenging to achieve [4,5]. Indeed, at a genetic level, both GPC and grain yield-related traits are determined by multiple quantitative trait loci (QTLs) interacting with each other and the environment. The negative correlation between GPC and yield-related traits has been observed in both segregating populations and germplasm collections [6,7]. Several studies have considered GPC and grain-yield components simultaneously assessed on the same population to identify GPC loci without pleiotropic effects and/or not closely linked to gene for low yield-related traits, and interesting results were reported both for 2A [8,9,10,11,12,13] and 2B chromosomes [12,14,15,16,17]. The identification of genetic sources of elevated protein content without negative pleiotropic effects would indeed be useful for improving GPC and GY simultaneously.

Improving GPC could be pursued by considering a candidate gene approach. Taking advantage of the recent advances in both bread and durum wheat genome sequencing and annotations [18,19], the identification of genes playing a key role in the Nitrogen Use Efficiency (NUE) process, such as N uptake from the soil, amino acid metabolism, or N transferring to the protein in the grain, has become an effective approach.

The glutamine synthetase (GS)/glutamate synthase (GOGAT) cycle represents a bottleneck in the first step of nitrogen assimilation, as these two enzymes work synergistically to incorporate the up-taken ammonium into organic molecules [20].

QTLs for GPC and NUE have been located on all chromosomes, and several authors reported major ones on 2A and 2B chromosomes of bi-parental mapping population, where *GS2* and *Fd-GOGAT* genes have been mapped [8,9,10,11,14,15,16]. Nevertheless, QTLs validation is of primary importance for further breeding programs development or gene cloning. Single QTL effect on phenotype might be population-specific and/or overestimated, and this has been demonstrated for complex traits such as GPC [15]. The constitution of near-isogenic lines (NILs) for the two different alleles of a target QTL is a useful and efficient method to validate a putative QTL. NILs are lines segregating only at target QTL but homozygous at the rest of the genome. Principally, NILs can be obtained by two approaches: (a) by backcrossing lines carrying the QTL region from a donor to a receipt line several times [21,22]; and (b) by exploiting the residual heterozygous lines (RHLs) from inbred populations and generating heterogeneous inbred family (HIF) at a QTL region [23,24], with the latter being more efficient and more rapid for both major and minor QTL [25].

In previous studies, we identified *GS2* and *Fd*-*GOGAT* genes as good candidates for GPC QTL with no pleiotropic effect on yield in durum wheat on 2B and 2A chromosomes, respectively [11,15,26,27]. Functional markers based on polymorphisms detected within the sequences of the two genes in the two parental lines were mapped in the durum wheat Svevo × Ciccio (S×C) RILs population [28]. *GS2* gene co-localized with a GPC-QTL detected on chromosome arm 2BL, in the region flanked by the markers D_304657 and Xwmc332, significant in one environment and across environments with a LOD ≥ 3.0 [26]. *Fd-GOGAT* gene co-localized with a GPC QTL detected on chromosome arm 2AS, in the region comprised between the markers Xgwm372c and the EST-SSR TC82001 (including the two closer markers Xgwm339 and Xgwm95), significant in two environments and across environments. The Svevo allele (increasing the trait) had positive additive effects ranging from 0.13 to 0.27 with a mean R^2^ value of 0.24, and the percentage of phenotypic variance explained by the additive effects of the mapped QTL ranged from 6% to 19.4% between environments and the mean was 19.4% across environments [27].

QTL analysis performed with CIM (Composite Interval Mapping) confirmed the presence of these markers in two major QTL for grain protein content. Recently, these data were confirmed on a wider durum wheat collection consisting of 236 durum wheat genotypes [29,30]. In addition, we found a significant difference in both *GS* gene expression and enzymatic activities between the durum wheat *cvs* Ciccio and Svevo, consistently higher in the last one [31]. Following up these results, here we present the development of two distinct set of heterogeneous inbred family (HIF)-based NILs segregating for *GS2* and *Fd-GOGAT* genes from heterozygous lines at those loci (previously identified in the S×C RIL population), and their genotypic and phenotypic characterizations, aimed to validate the previously identified GPC QTL on 2A and 2B chromosomes. Furthermore, we investigated the genomic characterization of the promoting regions of *GS2* and *Fd-GOGAT* genes, as regulatory elements involved in the transcription processes might be the ones responsible for the different level of gene expression and might contribute in assessing the role of these key genes in GPC control.

## 2. Results

### 2.1. Development of NILs at the GPC QTL on Chromosomes 2A and 2B

The S × C mapping population as previously phenotyped for GPC and used to both genetically and physically map *GS2* and *Fd-GOGAT* genes [26,27]. The RIL population consisted of 120 lines, two of which were found to be residual heterozygous lines at *GS2* and *Fd-GOGAT* genes, respectively, and selected to develop the two NIL sets. Specifically, the RIL SC45 F6:F7 was found to be heterozygous for *GS2* gene, and thus was allowed to self-pollinate to F8:F9 generation to develop *GS2*_NIL lines. The resulting 12 lines were tested with the previous reported functional marker GS-46 [26], as well as with four additional flanking markers from the integrated SNP map S×C [32] and six of them were excluded as still heterozygous at the locus. The final *GS2*_NIL genotypes consisted of six lines, three being homozygous for the high GPC allele of parental line Svevo (SC45-2, SC45-7 and SC45-12) and the remaining homozygous with the low GPC allele of parental line Ciccio (SC45-3, SC45-6 and SC45-11). Likewise, the *Fd-GOGAT*_NIL population was obtained from line SC107, heterozygous for *Fd-GOGAT* gene (Table 1). Although in this case the marker was dominant, the homozygosity and heterozygosity were confirmed by analyzing each line’s progeny. In addition to the GS2 NILs lines, four additional flanking markers from the integrated SNP map S×C [32] were used to genotype the *Fd*-*GOGAT* NILs. Out of 15 plants achieved from its self-pollination, only two were homozygous for the parental Ciccio allele (low GPC) (SC107-9 and SC107-15). Two lines homozygous for the parental Svevo allele (high GPC) were chosen among the available 13, based on their homozygosity at co-segregating markers (data not shown). The final *Fd-GOGAT*_NIL population consisted then of four lines. Nevertheless, one of them, SC107-15, was lost as no seeds were produced, therefore at the end the *Fd-GOGAT* NIL set comprised three lines, with two, SC107-1 and SC107-8, carrying the Svevo allele.

Microsatellite marker Xwmc332 and several SNP markers from the Svevo × Ciccio map [32] were analyzed in the NILs and this allowed us to reduce the GPC QTL on 2B to 4.7 cM. For QTL on 2A chromosome, EST-SSR TC82001 and several SNP markers were used to analyze the NILS, reducing the GPC QTL region to 4.9 cM. Those QTL interval regions were searched in the Svevo Genome Browser, and the gene lists within them were downloaded and screened. Candidate genes potentially involved in nitrogen and ammonium metabolism (genes that might play a key role in the N uptake from the soil, and of those controlling the enzymes of amino acid metabolism, potentially involved in N transferring to the protein in the grain) were looked for, but none was found.

### 2.2. Phenotypic Performance and Variation of the HIF Families

*GS2*_NIL and *Fd-GOGAT*_NIL lines were grown in five different site × season environments, and GPC, GYS and TKW data retrieved.

Concerning *GS2*_NIL, significant differences in GPC among parental lines and each derived NIL family were observed among the trials conducted in different locations, while Tukey Pairwise Comparisons showed a more variable situation for both GYS and TGW means of parental lines and NILs, most likely due to the environmental factors affecting those yield-related traits. Svevo had significantly higher GPC than Ciccio in all environments as well as the derived heterogeneous inbred family. Indeed, considering the mean across environments, the two parental lines showed a significant difference in their GPC values, as well as their derived NIL lines (SC45-2, SC45-7, SC45-12, and Svevo grouped together, as well as SC45-3, SC45-6, SC45-11, and Ciccio) (Table 2). Interestingly, the data also show a different situation for both GYS and TGW means, as no significant differences could be observed either between the two parental lines or their derived NILs sets, thus being a positive result considering the strongly negative correlation between GPC and yield.

The same situation was observed when considering ANOVA and Tukey’s comparison of *Fd-GOGAT* HIF for GPC, GYS, and TKW. As seen for the *GS2* HIF, Svevo had significantly higher GPC than Ciccio in all environments as well as its derived heterogeneous inbred family over the ones from Ciccio. Table 3 shows that, considering the mean across environments, SC 107-1 and SC 107-8 had higher GPC values, as well as Svevo. On the other side, SC 107-9 showed lower values, similar to the parental line Ciccio. No significant differences could be observed either between the two parental lines or their derived *Fd-GOGAT* HIF for GYS and TGW means across environments.

Data of yield of 1-m row were also analyzed to estimate yield on an area basis for both NILs groups. Within GS2 HIF derived NILs, values ranged from 0.78 (lineSC45-2) to 0.90 kg/m^2^ (line SC45-7). Likewise, Within Fd-GOGAT HIF derived NILs, values ranged from 0.53 (line SC107-1) to 0.63 kg/m^2^ (line SC107-9). No significant statistic difference was observed within the NILs.

### 2.3. Sequencing of the GS2 Gene Promoter Region

*GS2* gene structure and characterization have been reported in both durum and bread wheat [26,31,33]. Both A and B homoeologous genes are organized into 13 exons and 12 introns, with two alternative splicing forms, while the D genome homoeologous has just one transcript form and one more exon at 5′ terminus, which is a UTR region (http://plants.ensembl.org/Triticum_aestivum/).

**Figure 1:**
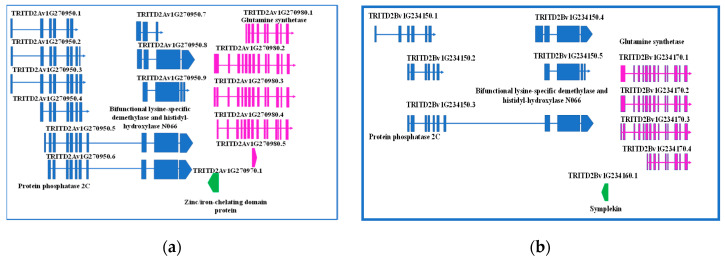
Detailed representation of genes located upstream *GS2-2A* (**a**) and *GS-2B* (**b**) genes, their structures, function, and eventual splicing isoforms.

The fine investigation of this region was performed by searching the durum wheat genome browser, *T. durum* (Svevo.v1) [19]. The first exon of *GS2-A2* gene was found to overlap with another element, a single exon gene encoding a zinc/iron-chelating domain protein G, oriented in the minus strand. Interestingly, upstream this gene, another one, having up to nine different splicing forms, was found, which encodes for a protein phosphatase 2C, a lysine-specific demethylase NO66, or a bifunctional lysine-specific demethylase and histidyl-hydroxylase NO66, depending on the splicing form. The region was also screened for regulatory elements, but none was found (Figure 1a).

A 333-bp element was found in the minus strand upstream *GS2-B2* gene, but it was not overlapping. Sequence analysis determined it is a member of *Symplekin* gene family, which encodes a nuclear protein involved in the regulation of polyadenylation and promoting gene expression. As reviewed by Hunt (2008) [34], the protein forms a high-molecular weight complex with components of the polyadenylation machinery. It is thought to serve as a scaffold for recruiting regulatory factors to the polyadenylation complex. It also participates in 3’-end maturation of histone mRNAs, which do not undergo polyadenylation. As reported for the region upstream *GS2-2A* gene, also on the B genome a gene having five different splicing forms was found, encoding either for a protein phosphatase 2C or bifunctional lysine-specific demethylase and histidyl-hydroxylase NO66, depending on the splicing form.

Interestingly, a region of 160 bp of the *Symplekin* gene was identified as a MITE element. In addition, 312 bp upstream this region, a 94-bp conserved element was found, which has been identified several times in coding regions of bread wheat, once reported as ncRNA (Figure 1b).

Two sets of genome specific primers were then designed and used to amplify the genomic region upstream *GS2* homoeologous genes in the two durum wheat parental lines Svevo and Ciccio and the HIF-NIL lines (primer sequencing and amplification condition are reported in Table S1).

**Figure 2:**
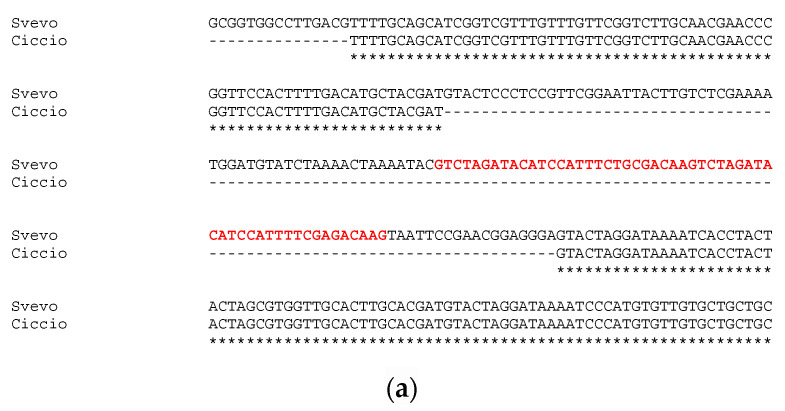

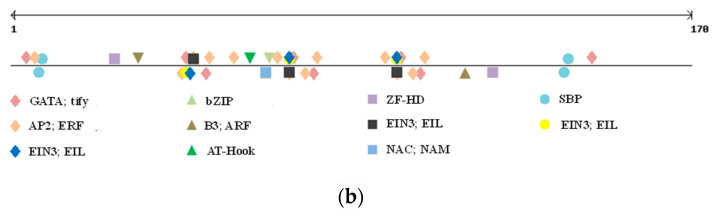
Alignment of *GS2B* gene’s promoter sequenced in Svevo and Ciccio *cvs.* with the tandem repeat reported in red bold (**a**); and details of TF families’ position found in the indel (**b**).

Sequencing of approximatively 1 kb upstream the first exon of *GS-A2* gene revealed an identity of 100% between Svevo and Ciccio sequences, with no gaps or single nucleotide polymorphisms detected. Instead, the sequencing and analysis of *GS-B2* gene promoter region highlighted a slightly different situation. The alignment of *GS2-B2* gene promoters of Svevo and Ciccio revealed a 132-bp deletion in Ciccio *cv* (Figure 2a). The deletion in Ciccio *cv* entailed the loss of 83 bp of the previously reported conserved element, including a 55 bp tandem repeat, whose 27-bp seed was repeated twice.

The polymorphic region was screened for eventual TFBSs, as well as GpC islands and repetitive motifs by searching PlantPAN3.0 database for *A. thaliana*, *Oryza sativa*, *Brachypodum distachyon*, and *Zea mais.* While no GpC island was located in the polymorphic indel, a number of TFBSs were detected (Figure 2b).

We only considered TFBSs having a perfect match with the ones already reported in the abovementioned species (score = 1). As shown in Table 4, nine different TFBSs were detected within the indel sequence, and all of them, except for the SBP binding factor, were located within the tandem repeat region. Positive regulator to ethylene response pathway, zinc finger domain, and bZIP binding sites were found, but the most interesting one was represented by a NAC; NAM binding site. Indeed, while all the other TFs binding to the identified sites are involved in generic plant stress response and metabolism, this specific one has been reported as affecting grain protein content and micronutrient amount [35,36,37,38].

### 2.4. Sequencing of the Fd-GOGAT Gene Promoter Region

*Fd-GOGAT* gene structures have been extensively reported in durum wheat [27]; this large gene (33 exons and 32 introns) spans over about 15 kbp. Moreover, several different splicing isoforms have been found for homoeologous genes, as well as precisely four for 2A and eight for 2B homoeologous chromosomes. The regions upstream *Fd-GOGAT* genes were investigated to detect eventual close and/or overlapping genes in the promoter regions as observed for the *GS2* genes, but none was found.

As done for *GS2* genes, a set of genome specific primer was also designed to amplify and sequence both *Fd-GOGAT-2A* and *Fd-GOGAT-2B* promoters in Ciccio, Svevo, and their derived HIF-NILs lines. While no polymorphism was detected for the 2B promoter, some were found in the 2A homoeologous gene. Specifically, three SNPs were detected between *cv* Ciccio and Svevo: two transversions (G:T and C:G) and one transition (A:G). The promoter regions were investigated and screened in both parental lines for eventual differences in TFBS sites due to the nucleotide polymorphisms mentioned above, and some interesting differences were outlined, as shown in Table 5. The detected SNPs determined the occurrence or loss of some specific TFBSs at that site, resulting in additional BSs or, in some cases, in specific and unique BSs for one of the two parental lines. Specifically, in *cv* Svevo, a TCP and a Myb/SANT; G2-like TFBSs were detected at high similarity score (>0.9) in the regions where SNPs were located. Moreover, these polymorphisms determined the occurrence of additional TFBSs to those already reported for both *cvs*, and specifically a Trihelix, a bHLH, and a NAC; NAM site.

A gene group analysis performed simultaneously on Svevo’s *GS-B2* and *Fd-GOGAT_2A* promoter regions determined the co-occurrence of a number of TFBSs. Specifically, 252 common sites were identified, with most of them belonging to specific TF families, such as AP2, ERF, bHLH, bZIP, Dof, MYB, NAM, and WRKY.

## 3. Discussion

Here, we report the development of two distinct sets of HIF-based NILs, segregating for *GS2* and *Fd-GOGAT* genes, from heterozygous lines at those loci, previously identified in a durum wheat RIL population, as well as their genotypic and phenotypic characterization.

The HIF method [24] used in this work allowed us to generate useful NILs at a single locus from a single cross (RIL Svevo × Ciccio [28]) instead of using markers flanking a QTL [39]. Single functional markers, for *GS2* and *Fd-GOGAT* genes, respectively, both linked with the GPC QTL of interest, were used for generating the two NILs sets. Compared to the traditional method, which is based on the selection of two flanking markers delimiting a QTL of interest, the HIF method allows the production of NILs with reduced sizes of the “non-desirable” chromosomal segment discriminating the isolines.

Traditionally, each NIL set has its own genetic composition, which determines unique morphological and phenological characteristics influencing the expression of a quantitative trait [38].

The assessment of the effect of a particular gene on a specific trait should be more accurate by using a NIL pair obtained by the HIF method, as fewer isolines would be needed for investigating the effect of a QTL/gene.

Thus far, HIF-based NILs have been intensively used for investigating the effects of various QTL and traits of interest in wheat, such as dormancy QTL [40], pre-harvest sprouting [41], and spikelet number per spike [42].

During the past decades, the increase in GPC has mainly been achieved by intensifying nitrogen (N) fertilization. Considering the high costs of N fertilizers and the harmful environmental impacts of nitrate loss from the soil, decreasing the amount of N applied to cereal cropping systems while maintaining high productivity of modern cultivars has therefore become a breeding priority. The identification of candidate genes and their allelic variants affecting this trait is an effective method to develop new wheat varieties with high GPC. Nitrogen remobilization efficiency is an important factor increasing grain protein content (GPC) and consequently, the nutritional and technological properties of flour and semolina [43,44]. Several authors have focused on deciphering GPC and NUE QTLs, and genetic diversity at candidate genes have recently been considered for this purpose. Indeed, in our previous studies, we identified both *GS2* and *Fd-GOGAT* genes as good candidates in GPC study in the S×C RIL population [26,27]. Specifically, these two genes work synergistically in the GS/GOGAT cycle, which has a key role in nitrogen assimilation and recycling in young leaves within the chloroplast, where nitrite reduction occurs, and ammonium is assimilated [45]. Both genes are located on the chromosome 2 homoeologous group, whose influence on GPC control was reported in different genetic materials, thus suggesting its key role in the control of the character [11,15,46,47,48].

Interestingly, Kichey et al. (2005) and Habash et al. (2007) [9,49] showed that QTLs for GS activity co-localized with QTL for grain N on chromosome 2A and hinted this may be coincident with QTL on 2B and 2D homeologs for soluble protein content, and that increased activity was associated with higher grain N. This finding was confirmed on different genetic material [50]. In addition, in maize, the *GS2* locus was found to be coincident with a leaf senescence QTL [51], but, unlike in maize, Fontaine et al. (2009) [50] did not find a correlation between GS activity and yield components in wheat, this being in agreement with our data. Indeed, as previously reported [11,15], QTL for GPC were found on 2A and 2B chromosomes, in the same region where *Fd*-*GOGAT* and *GS2* gene were mapped in Svevo × Ciccio RIL population, respectively [26,27]. Both authors reported that these QTLs showed no negative effects on grain yield related traits, making them good candidate for marker-assisted selection to improve GPC and grain yield simultaneously. However, considering that a QTL usually spans several cM, it is necessary to perform more detailed genetic analyses on larger populations or specific genetic material, such as NILs, in order to assess whether a single gene with pleiotropic effects or different loci within the linkage group are responsible for the different traits. The near-isogenic lines we developed at both *GS2* and *Fd-GOGAT* loci showed that, despite having significant differences in GPC, no significant ones were observed in GYS. This could be either explained with both *GS2* and *Fd-GOGAT* having no negative pleiotropic effects on yield components, or that a potential effect is actually masked by environment. Despite in both HIF-NILs families it was noticed that lines having the Svevo allele showed higher GPC, it was also outlined that the differences observed within NILs were highly statistically significant especially for *GS* HIF-derived NILs, as *Fd-GOGAT* HIF-derived ones showed a lower value of significant difference, as shown in Table 2 and Table 3. In both cases, we could assume that the Svevo allele is the one increasing the GPC trait.

The differences in GPC between the isolines developed for two key genes involved in nitrogen metabolism *Glutamine synthetase* and *Glutamate synthase,* not only further confirm the significance of these two genes in the processes determining the final grain protein content but would also facilitate the exploitation of these HIF NILs families in further characterizing the major GPC QTL on 2A and 2B chromosomes, studying their interaction with other traits of agronomic importance, and developing functional markers that can be reliably used to follow these major loci.

For *GS2* genes, a different gene expression was reported between the two cultivars that could affect the GPC content [31]. In order to detect eventual polymorphism potentially involved in a differential gene expression, the promoter regions of both *GS2* and *Fd-GOGAT* genes were screened in Svevo and Ciccio parental lines.

Among all regulatory elements, Transcription Factors (TFs) regulate cell processes by binding a specific DNA motif on promoter regions and affecting downstream gene expression.

By comparing the promoting regions of the genes considered in this study, the most interesting result was observed in the promoting region of *GS-B2* gene, as a 132-bp indel was detected between the two parental lines. The analysis of this region outlined the presence of a 55-bp tandem repeat, as well as a number of TFBSs, such as EIN/EIL1, bZIP, AP2/ERF, SPL, ZF-HD, and NAC; NAM.

A recent review reported the role of EIN3/EIL1 transcription factors in *Arabidospis*, highlighting their key role as regulators of ethylene signaling [52]. Ethylene regulates many different aspects of plant development and stress responses, thus its signaling pathway needs proper modulation depending on the plant conditions. Salih et al. (2020) [53] reported a very recent study on the possible functions of EIL/EIN3 proteins in cotton. Their GO annotations and KEGG pathway analyses indicated that, besides being involved in the ethylene-activated signaling pathway and a number of cellular macromolecule metabolic process, a great number of EIL/EIN3 genes were also involved in nitrogen compound metabolic process.

In addition, a bZIP binding site was found in the polymorphic indel, which represents one of the largest and most variable families of TFs and are uniquely present in eukaryotes. A genome-wide analysis and gene ontology enrichment analysis of bZIP Transcription Factors performed on 191 bZIP TFs, identified in bread wheat, reported that some of them are involved in cellular metabolic processes related to nitrogen compounds [54]. More investigations will be needed to define and characterize the interaction between these TFs and *GS2* gene in order to better explain the role of these TF families in nitrogen metabolism pathway.

AP2/ERF transcription factors were classified into five subfamilies [55], the most known of which are: AP2 (APETALA2), RAV (related to ABI3/VP1), DREB (dehydration-responsive element binding protein), and ERF (ethylene-responsive factor). The AP2/ERF transcription factors were shown to regulate diverse processes of plant development and stress responses, such as vegetative and reproductive development, cell proliferation, abiotic and biotic stress responses, and plant hormone responses [56,57,58].

*SQUAMOSA Promoter-Binding Protein-Like* (SPL) genes have been shown to play numerous important roles during plant growth and development [59]. SPLs are known to regulate several biological processes, including leaf development [60], phase transition [61], flower and fruit development [62], plant architecture [63], sporogenesis [64], GA signaling [65], and response to copper and fungal toxin [66,67].

Abu-Romman (2014) [68] reviewed ZF-HD TF family and reported that these classes of homeodomain proteins are involved in regulating intercellular trafficking [69], inflorescence stem growth [70], and hormone and stress signaling [71,72,73].

Among all TFBSs identified within the indel in the *GS2* gene promoter in *cv* Svevo, the NAC; NAM TFBS was the most interesting and potentially involved in the different expression of *GS2* gene and final GPC between the two analyzed *cvs*. Several studies have reported that wheat NAC TFs are involved in several biological processes such as senescence and nutrient remobilization [35,74], and stress response, both to biotic (such as stripe rust [75,76,77,78]) and abiotic stresses including drought and salt tolerance [79,80,81,82,83]. Specific studies have been carried out on NAM TFs in relation to GPG and nitrogen metabolism. Waters et al. (2009) [84] reported a *TaNAM* gene which increased protein content in the grain by increasing the remobilization of nitrogen from vegetative tissues. Interestingly, He et al. (2015) [85] described a *TaNAC2* TF that positively regulated *TaGS2* expression.

In conclusion, the data reported in the present work confirm that *GS2* and *Fd-GOGAT* genes are involved in grain protein accumulation and suggest that the surrounding genomic regions and their promoters could affect gene expression. The identification of new useful superior alleles for both genes could be employed for marker-assisted selection and the constitution of wheat varieties with improved agronomic performance and N-use efficiency.

## 4. Materials and Methods

### 4.1. Plant Material

The Svevo × Ciccio mapping population, consisting of 120 F6:F7 RILs [28], was considered in previous studies to genetically dissect important agronomic traits, such as grain yield components, grain protein content, and yellow pigments [11,31]. We focused on data related to GPC, and, specifically, on RILs which have been shown to have residual heterozygosity at *GS2* and *Fd-GOGAT* candidate genes. Near isogenic lines were indeed obtained starting from heterozygous lines at the genotype of the marker associated with the QTL of interest in the F6 RILs, SC45 and SC107, heterozygous at *GS2* and *Fd-GOGAT* loci, respectively. The two set of heterogeneous inbred families (HIF) were generated by self-crossing to F8:F9 generation and subsequently used to validate individual effects of the putative QTLs in different environments.

### 4.2. Experimental Design and Phenotypic Evaluation

The two NILs sets, containing heterogeneous inbred families, respectively, three for *Fd-GOGAT* and six for *GS2* genes, as well as the two parental lines Svevo and Ciccio, were grown for three growing seasons (2012–2013, 2013–2014, and 2014–2015) at the experimental field trials of Bari University, Valenzano (Bari), and Policoro (Potenza), both located in South Italy. Field trials were organized as a randomized complete blocked design (RCBD) for all entries, with ten total blocks, each including all lines, and each line was sown in a plot of one m^2^. In total, 14 genotypes were tested including parents as double check, for ten replicas (140 plots) each year. Standard agronomic practices were followed, and, during the growing season, 80 kg/ha of N were applied. At maturity, plots were hand harvested and phenotypic data and measurements were recorded for grain protein content (GPC), grain yield per spike (GYS) and thousand kernel weight (TKW). Grain protein content, expressed as a percentage of protein on a dry weight basis, was determined on 3 g sample of whole-meal flour using near-infrared reflectance spectroscopy (Spectra Alyzer Premium, Zeutec Büchi, Rendsburg, Germany). For each plot, the plants were harvested, seeds collected, five replicas analyzed, and a mean value given as line value. TKW was evaluated on a 15g seed sample per plot.

### 4.3. Statistical Analysis

The data were analyzed as a randomized design with three biological and five technical replicates and expressed as means ± SE. Statistical analysis was carried out using Sigma Plot software 12.0 (Systat Software, Inc., San Jose, CA, USA). One-way analysis of variance (ANOVA) and Tukey’s comparison test were used to calculate the difference between the genotypes and NILs families. Differences were considered statistically significant at a *p*-value of < 0.05.

### 4.4. Molecular Marker Analysis

Molecular analysis was carried out to confirm the segregation at loci of interest and to check for casual contaminations. DNA was extracted from leaves collected at tillering stage from both parental lines and NILs using a modified CTAB method. The chromosomal location of *GS2* and *Fd-GOGAT* gene functional markers, along with their genetic distances on the durum wheat map, their sequences, and PCR conditions, are, respectively, reported in [26,27].

### 4.5. Promoter Region Sequencing

The promoter regions of both 2A and 2B homoeologous *GS2* and *Fd-GOGAT* genes were retrieved from both bread and durum wheat genomes (*cvs* Chinese Spring and Svevo) (http://plants.ensembl.org/Triticum_aestivum/Info/Index, https://www.interomics.eu/durum-wheat-genome-intranet). Two sets of genome specific primer pairs were designed for each gene by using OligoExplorer software (http://www.genelink.com/tools/gl-oe.asp) and subsequently used to amplify both promoter regions in the durum wheat *cvs* Ciccio and Svevo (to exclude possible casual variety contamination) as well as in the two HIF-based NILs families, as previously reported [86]. Primer sequences are reported in Table S1. Single PCR fragments were cloned and sequenced as reported in [87].

The sequenced promoters of the two *cvs* were aligned and compared in order to detect possible polymorphisms potentially affecting transcription factors binding sites (TFBSs) by PlantPan3.0 database (http://plantpan.itps.ncku.edu.tw/index.html).

## Figures and Tables

**Table 1:** Generation of heterogeneous inbred family (HIF)-NILs by self-crossing RILs from the Svevo × Ciccio mapping population having residual heterozygosity at *GS2* and *Fd-GOGAT* candidate genes, respectively. In bold, the selected lines from which (HIF)-NILs were developed.

Line	Generation	Gene Marker Profile
**SC45**		**GS-2B**
Svevo (A)	F8:F9	507 bp
Ciccio (B)	F8:F9	473 bp
SC45-1	F8:F9	H
**SC45-2**	F8:F9	**A**
**SC45-3**	F8:F9	**B**
SC45-4	F8:F9	H
SC45-5	F8:F9	H
**SC45-6**	F8:F9	**B**
**SC45-7**	F8:F9	**A**
SC45-8	F8:F9	H
SC45-9	F8:F9	H
SC45-10	F8:F9	H
**SC45-11**	F8:F9	**B**
**SC45-12**	F8:F9	**A**
**SC107**		**Fd-GOGAT-2A**
Svevo (A)	F8:F9	(284, 290) bp
Ciccio (B)	F8:F9	(-, 290) bp
**SC107-1**	F8:F9	**A**
SC107-2	F8:F9	A
SC107-3	F8:F9	A
SC107-4	F8:F9	A
SC107-5	F8:F9	A
SC107-6	F8:F9	A
SC107-7	F8:F9	A
**SC107-8**	F8:F9	**A**
**SC107-9**	F8:F9	**B**
SC107-10	F8:F9	A
SC107-11	F8:F9	A
SC107-12	F8:F9	A
SC107-13	F8:F9	A
SC107-14	F8:F9	A
**SC107-15**	F8:F9	**B**

**Table 2:** Mean values for Grain protein content (GPC), grain yield per spike (GYS), and thousand grain weight (TGW) of heterogeneous inbred family (HIF)-based NILs segregating for *GS2* and parental lines Svevo and Ciccio evaluated in five different environments. Different letters indicate significant differences (one-way ANOVA and Tukey’s tests; *p* < 0.05).

	*GS2 HIF*			
GENOTYPE	ALLELE	GPC	GYS	TGW
SC45-2	Svevo	15.48 ^A^	1.95 ^AB^	41.24 ^A^
SC45-7	Svevo	15.20 ^A^	2.25 ^A^	43.72 ^A^
SC45-12	Svevo	15.27 ^A^	1.98 ^AB^	42.92 ^A^
SC45-3	Ciccio	13.61 ^B^	1.98 ^AB^	44.55 ^A^
SC45-6	Ciccio	13.82 ^B^	2.01 ^AB^	45.30 ^A^
SC45-11	Ciccio	13.25 ^B^	2.04 ^AB^	45.68 ^A^
Svevo		15.25 ^A^	1.91 ^AB^	41.46 ^A^
Ciccio		13.02 ^B^	1.74 ^B^	43.58 ^A^

**Table 3:** Mean values of Grain protein content (GPC), grain yield per spike (GYS), and thousand grain weight (TGW) of heterogeneous inbred family (HIF)-based NILs segregating for *Fd-GOGAT* and parental lines Svevo and Ciccio evaluated in five different environments. Different letters indicate significant differences (one-way ANOVA and Tukey’s tests; *p* < 0.05).

	*Fd-GOGAT HIF*			
GENOTYPE	ALLELE	GPC	GYS	TGW
SC 107-9	Ciccio	13.72 ^BC^	1.57 ^AB^	42.21 ^A^
SC 107-1	Svevo	14.32 ^AB^	1.32 ^B^	41.21 ^A^
SC 107-8	Svevo	14.40 ^AB^	1.53 ^AB^	43.42 ^A^
Svevo		15.25 ^A^	1.91 ^AB^	41.46 ^A^
Ciccio		13.02 ^B^	1.74 ^B^	43.58 ^A^

**Table 4:** Transcription factor (TF) binding sites detected in the polymorphic indel between the two parental lines and NIL families for *GS-B2* gene.

TFBSs	Hit Sequence	Strand	Corresponding TF (Gene ID)	Description
EIN3; EIL	aTGTATcta	+	AT2G27050	Ethylene insensitive 3
	tagATACAt	−		
	tagATACAt	−		
	ggATGTAtcta	−	Os09g0490200	Ethylene insensitive 3
	tagaTACATcc	+		
	tagaTACATcc	+		
	gaTGTATcta	−	AT5G21120	Ethylene insensitive 3
	tagATACAtc	+		
	tagATACAtc	+		
NAC; NAM	TACGT	−	AT1G01720	No apical meristem (NAM) protein
AT-Hook	aAAATAc	+	Os01g0835600	AT hook motif
GATA; tify	GGATG	+	AT1G51600	GATA zinc finger
	TATCT	−		
	AGATA	+		
	CATCC	−		
	GTATC	−		
	GATAC	+		
bZIP	TACGT	−	AT3G54620	bZIP transcription factor
	ACGTC	+		
ZF-HD	ATTAC	+	AT1G75240	ZF-HD protein dimerization region
	GTAAT	−		
AP2; ERF	ATCTA	+	AT3G14230	AP2 domain
	TAGAT	−		
B3; ARF	TGTCTc	+	AT1G19220	auxin response factor
	gAGACA	−		
SBP	GTACT	+	AT2G33810	squamosa promoter binding protein-like 3
	AGTAC	−		

**Table 5:** Specific and additional Transcription factor (TF) binding sites detected in parental line Svevo and its derived *HIF* for *Fd-GOGAT* gene due to SNPs polymorphism in the promoter region.

TF Family	Hit Sequence	Strand	Corresponding TF (Gene ID)	Description
TCP	CGGGT	−	AT3G27010	Anatomical structure morphogenesis
Myb/SANT; G2-like	acaGAATAaagacaa	−	AT1G32240	Homeodomain-like superfamily protein; Probable transcription factor KAN2
Trihelix	aAAATAc	+	AT5G01380	Homeodomain-like superfamily protein; Trihelix transcription factor GT-3a
bHLH	CACATG	+	AT1G32640	Basic helix-loop-helix (bHLH) DNA-binding family protein; Transcription factor MYC2
NAC; NAM	CATGTG	+	NAC; NAM	No apical meristem (NAM) protein

## Supplementary Materials

Supplementary Materials can be found at https://www.mdpi.com/1422-0067/21/23/9253/s1. Table S1. Oligo name and sequences of primer combinations used to amplify promoter regions of *GS2* and *Fd-GOGAT* homoeologous genes.

## Abbreviations

GPC	grain protein content
GS	glutamine synthetase
GOGAT	glutamate synthase
NUE	Nitrogen Use Efficiency
NILs	near-isogenic lines
HIF	heterogeneous inbred family
