# Supplementary material for: Functional Validation of Glutamine synthetase and Glutamate synthase Genes in Durum Wheat near Isogenic Lines with QTL for High GPC
Source: Int J Mol Sci. 2020 Dec 4;21(23):9253. doi: 10.3390/ijms21239253 (PMC7730160; doi:10.3390/ijms21239253)
Supplement: Supplementary file 1 [file ijms-21-09253-s001.pdf]

Supplementary Table 1. Oligo name and sequences of primer combinations used to amplify promoter regions of *GS2* and *Fd-GOGAT* homoeologous genes.

**Oligo name**	**5'-3' sequence**
GS2_2A-1F	TTTGCCGAACAGTTTGTCCT
GS2_2A-1R	GAGCCAGAGGGAAGGAACAT
GS2_2A-2F	GTCCTGTACACACACTCCGA
GS2_2A-2R	GTTTCTGGACCGGTTTGCC
GS2_2A-3F	GTTCCATAGGGACATGGCGTG
GS2_2A-3R	GTCCTGTACACACACTCCGA
GS2_2A-4F	TCATATCCGGAGCCAGAGGGA
GS2_2A-4R	GGAGTAAGTAAGTAAGCAGCGG
GS2_2B-1F	TCAGTTCTTGTACA CACCG C
GS2_2B-1R	ACCTTTCAGCTGTTCACACGAA
GS2_2B-2F	ATTTCCGGATCAGTTCTTGTACA
GS2_2B-2R	CTGCAGCATAATCCCTGTAGC
GS2_2B-3F	ACCTTTCAGCTGTTCACACGAA
GS2_2B-3R	GTGTGTGCCTTCTTTGGACG
GS2_2B-4F	CTCTATCCGAGATGTGTGTGC
GS2_2B-4R	AAGGCGGCGGAGTAAGTAAG
GS2_2B-5F	AATAATATTTCCAGCGCGATCTC
GS2_2B-5R	CCCACCTGGCACTGCATC
Fd-GOGAT_2A-1F	TGACCGGCTGAATTCTACCA
Fd-GOGAT_2A-1R	CCTCCCCAATCCTGCAGAG
Fd-GOGAT_2A-2F	GACAAACGCACTCCTCATTCA
Fd-GOGAT_2A-2R	TACTCCTCTCCTCTCCTCCCCAC
Fd-GOGAT_2A-3F	TCCTCCCCACCGCAGCACC
Fd-GOGAT_2A-3R	AGGAGGTATGGCTCGCTCTG
Fd-GOGAT_2A-4F	CCCGCGCAGGAGGTATGGCTCGCT
Fd-GOGAT_2A-4R	GCTTGTGGTGTTGGGTTTGT
Fd-GOGAT_2B-1F	TGGAGAAGGAAAGGGGAAGG
Fd-GOGAT_2B-1R	ATGAGAAGCTTGACATGCCC
Fd-GOGAT_2B-2F	ACATATCACCATTCACCAGCC
Fd-GOGAT_2B-2R	GTTCTACCCATCAGCGCAAG
Fd-GOGAT_2B-3F	CAACACGCCAGCATCATCAA
Fd-GOGAT_2B-3R	GGCCCCTTTCCTGATCCAT
Fd-GOGAT_2B-4F	AATCACCCCTATACAAAACGTCA
Fd-GOGAT_2B-4R	GAAATCAAACCCAATCGCGAG
